# Infectious Diseases-Approved Virtual Reality Goggles for Common Procedures in Pediatric Surgical Patients

**DOI:** 10.3390/jcm13185572

**Published:** 2024-09-20

**Authors:** Yarden Gilboa Pras, Atara Indursky, Shai Gilboa Pras, Ortal Schaffer, Eleonora Niazov, Osnat Zmora

**Affiliations:** 1 Faculty of Medicine and Health, Tel Aviv University, Tel Aviv 6997801, Israel; yardenpras@gmail.com (Y.G.P.); atara.indursky@gmail.com (A.I.); shaigilboa1993@gmail.com (S.G.P.); ortal.schaffer@gmail.com (O.S.); gadi177@walla.co.il (E.N.); 2 Department of Pediatric Surgery, Shamir Medical Center, Zerifin 7073001, Israel

**Keywords:** virtual reality, pediatric surgery, infectious diseases

## Abstract

**Background/Objective**: Virtual reality (VR) technology has been reported as effective in mitigating fear, anxiety, and pain in children undergoing various medical procedures. Our aim was to test the effectiveness of non-sponge-coated mobile phone-operated VR goggles approved by infectious diseases (ID) control in pediatric surgical patients. **Methods:** A prospective randomized trial in which pre-procedural and post-procedural heart rate, fear, and anxiety, and post-procedural pain were measured in pediatric surgical patients assigned to receive standard care versus standard care and VR goggles. The patients underwent line insertions, peripheral venipunctures for blood draws, drain removals, and wound care. **Results:** The control group and the intention-to-treat group were each randomly assigned twenty-four patients. Since eight patients who received VR goggles removed them prior to completion of the procedure, the study group included sixteen patients. In the study group, heart rate, fear, and anxiety scores were significantly lower after the procedure as compared to prior to the procedure. In the control group, these parameters were similar before and after the procedure. Post-procedural decrease in fear and anxiety was significant when comparing VR to control. However, despite better post-procedural physiological and emotional scores in the VR group as compared to control, the differences were not statistically significant. Pre-procedural anxiety was significantly higher in the study group. **Conclusions:** ID-approved VR goggles can reduce fear and anxiety associated with procedures commonly performed in pedicatric surgical patients. However, since increased baseline anxiety might be associated with VR, a higher benefit might be achieved if goggles were worn only by children who express a clear desire to use them.

## 1. Introduction

The use of virtual reality (VR) technology to reduce pain, fear, and anxiety has been studied for various medical conditions, both chronic and acute. In children, successful use of VR goggles has been reported to reduce fear and anxiety and alleviate pain during various procedures. Some examples include cast removal [1], peripheral intravenous line insertion [2,3], replacing burn dressing [4,5], accessing port-a-cath in patients with oncological diseases [6], immunization [7], dental procedures [8,9,10], and more. VR is described as a fully digital experience that can either simulate or differ completely from the real world [4]. Studies which compared immersive VR to other distractvie techniques, such as video games [8], video watching or listening to music [9,10] found that immersive VR was more effective in alleviating pain and stress. It is thought that when the patient’s attention is distracted by VR, less attention will be given to pain perception [8,11,12,13].

The adverse events reported with using VR goggles are few and rare, including nausea [14], disorientation, disturbance in balance [15], and technical issues of fitting young children with goggles [16]. Children who are hospitalized and treated by pediatric surgery are usually more exposed to stressful and painful experiences. This leads to a high level of anxiety [17,18]. Higher levels of anxiety can in turn lead to higher levels of pain [19]. Therefore, there is a high need for effective methods to reduce pain, fear, and anxiety in pediatric surgical patients. To date, there are no reports on the use of VR goggles in the specific population of pediatric surgical patients undergoing various routine invasive procedures without sedation or general anaesthesia.

With the spread of the COVID-19 pandemic, the use of comfortable, high-quality VR goggles padded with sponge material has been discontinued in many places due to limitations in disinfecting the sponge surface between users and concerns about COVID-19 transmission. However, particularly during the COVID-19 pandemic, the use of virtual reality technology was found to be effective in various situations, ranging from interactions with isolated patients, reducing fear and anxiety in patients, medical education, and more [20]. VR goggles that are not sponge-coated can be more reliably disinfected between users and are therefore infectious disease (ID)-approved. However, these VR goggles are less comfortable and equipped with inferior technology.

In this study, we aimed to examine the effectiveness of non-sponge-coated VR goggles in reducing pain, fear, and anxiety during various procedures frequently required for pediatric surgical patients.

## 2. Materials and Methods

A prospective randomized study was conducted involving children aged 4–18 years between September 2021 and March 2024 with the following research objectives:

1. To assess the effectiveness of ID-approved VR goggles in reducing fear associated with common procedures performed in the pediatric surgical ward; procedures that are usually performed without sedation.
2. To assess the effectiveness of ID-approved VR goggles in reducing anxiety associated with the abovementioned procedures.
3. To assess the effectiveness of ID-approved VR goggles in reducing pain associated with the same procedures.

**Figure 1:**
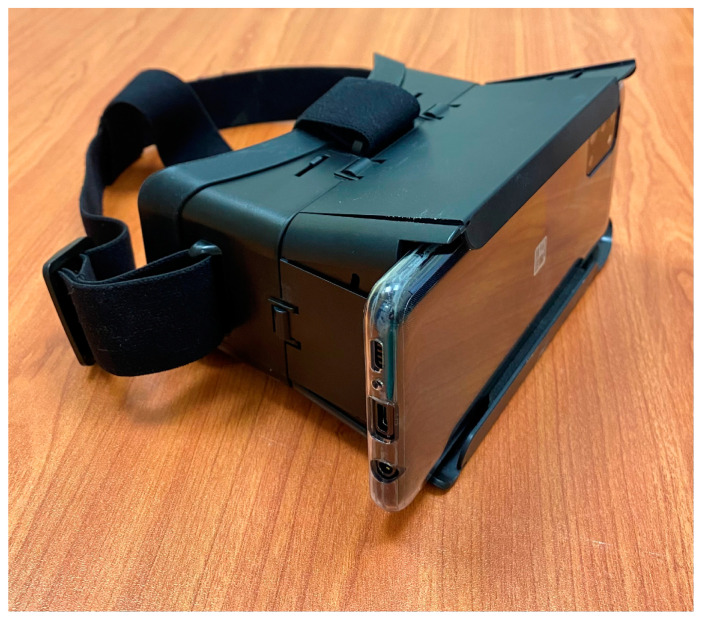
ID-approved VR goggles with smartphone docked.

After revision of various VR goggles, foam-coated models were not approved by the hospital’s ID team, despite previous reports of using disposable hygiene covers applied to the foam cover and wiping of the foam cover [1]. The only goggles approved were non-sponge-coated mobile VR headsets (VRyou technologies), with obligatory disinfection between users using quaternary ammonium wipes. The goggles were used with a Samsung Galaxy A31 smartphone inserted into a docking device (Figure 1). An immersive three-dimensional 360° audio-visual virtual experience of ocean diving was achieved by watching various sea creatures and other marine divers. By moving their heads, the children could explore the virtual environment surrounding them. Using the hand-held gaming controller was not desired as the upper arms needed to be guarded to prevent disruption of the medical procedure. Relaxing music was played without earphones. The length of the movie chosen for the study was 6:30 min, which allowed enough time for the procedure before completion of the video. One size of headset was used for all children. However, the headset was equipped with an adjustable inter-pupil distance and with adjustable straps which can fit various head sizes.

Patients hospitalized under the pediatric surgery service were invited to participate in the study if any of the three following procedures were about to be performed: blood sampling and IV-line insertion, drain removal, or wound care (irrigations for deep wound infection). After receiving verbal patient assent and written parent consent, patients were given their randomized group allocation: VR goggles (study group) versus control. Randomization of participants was performed using a computerized tool which created random number lists and was conducted separately for each of the three procedures. Standard care was applied in both study and control groups (explanation of the procedure in a comforting and supportive manner with the guardians and team being allowed to console and distract the patient). After measurement of heart rate and assessment of fear and anxiety levels, the participants donned the headset and the 3D movie was started. The medical procedure was then performed as the headset was worn and the 3D movie played througout the procedure. Immediately after completion of the procedure, the participants were asked to remove the headset. After removal of the headset, heart rate was measured, and pain, fear, and anxiety levels assessed. The following scales were used to asses emotional parameters:

1. Pain level—assessed immediately after the procedure using the Wong-Baker FACES Pain Rating Scale [21];
2. Fear level—assessed immediately before and after the procedure using the Children’s Fear Scale (CFS) [22];
3. Anxiety level—assessed immediately before and after the procedure using The Children’s Anxiety Meter-State (CAM-S) [23].

Demographic and clinical data collected included age, sex, diagnosis, and length of stay until procedure. Procedure-related data included: type of procedure, and number of prior similar procedures during the same admission.

Children suffering from psychosis or epilepsy or from psychiatric disorders that may be exacerbated in the VR environment (such as hallucinations), children with facial or respiratory infections, and children with severe visual impairment or blindness were excluded from participation.

The study was approved by the institutional Helsinki committee (approval # 0085-21-ASF).

### Statistical Analysis

Descriptive statistics in terms of mean, standard deviation, median, interquartile range, and percentages were calculated for all study parameters. Normal distribution of continous parameters was tested by the Kolmogorov–Smirnov test. As a result of this test, we used the *t*-test, Mann–Whitney U test, and Kruskal–Wallis test to compare continuous parameters between study and control groups. For comparison of categorical parameters between study and control groups, we used the Fisher exact test or Pearson chi square. Pearson correlation was used to test the relations between pre-procedural and post-procedural physiological and emotional parameters in the study and control groups. Repeated measure analysis was used to test changes at two time points (before procedure and after procedure) in physiological (heart rate) and emotional (fear and anxiety) parameters and to compare these changes between groups. A multivariate test was used to predict post = pre procedural changes in parameters with adjustments for demographic, clinical, and procedure-related characteristics. A multivariate general linear model was applied to predict pre-procedural and post-procedural heart rate, fear, anxiety, and pain with adjustments for demographic, clinical, and procedure-related characteristics; *p* < 0.05 was considered as significant. SPSS version 28 (IBM Corp, Armonk, NY, USA) was used for all statistical analyses.

## 3. Results

Over the study period, 48 children were randomized and included in the study. After obtaining consent, 24 children were allocated to standard care only (control group), and 24 to receive standard care and VR goggles. Eight participants allocated to receive the VR goggles removed them prior to starting the procedure or during the procedure, sometimes to be able to see what was going on during the procedure. Therefore, only 16 participants retained the goggles throughout the procedure (study group). Table 1 presents the clinical, demographic, and procedure-related characteristics of the control and study groups. The study group included a significantly higher proportion of female participants as compared to the control group (*p* = 0.04).

### 3.1. Comparison of Physiological and Emotional Parameters before and after Procedures in Each Group

In the control group, there were no significant changes in any of the parameters tested after the procedure as compared to prior to the procedure. However, in the study group, all parameters tested (heart rate, fear, and anxiety scores) were significantly lower after the procedure as compared to prior to the procedure (Table 2).

### 3.2. Comparison of Physiological and Emotional Parameters before and after Procedure between the Different Groups

Heart rate, pain, fear, and anxiety scores before and after the procedure were compared between the control group and the study group. Pre-procedural anxiety scores were significantly higher among the study as compared to control participants (*p* = 0.034). All parameters tested after the procedure were lower among participants who used the goggles as compared to control; however, the differences were not statistically significant (Table 3).

### 3.3. Comparison of Change in Physiological and Emotional Parameters Following Procedure in the Study and Control Groups

**Figure 2:**
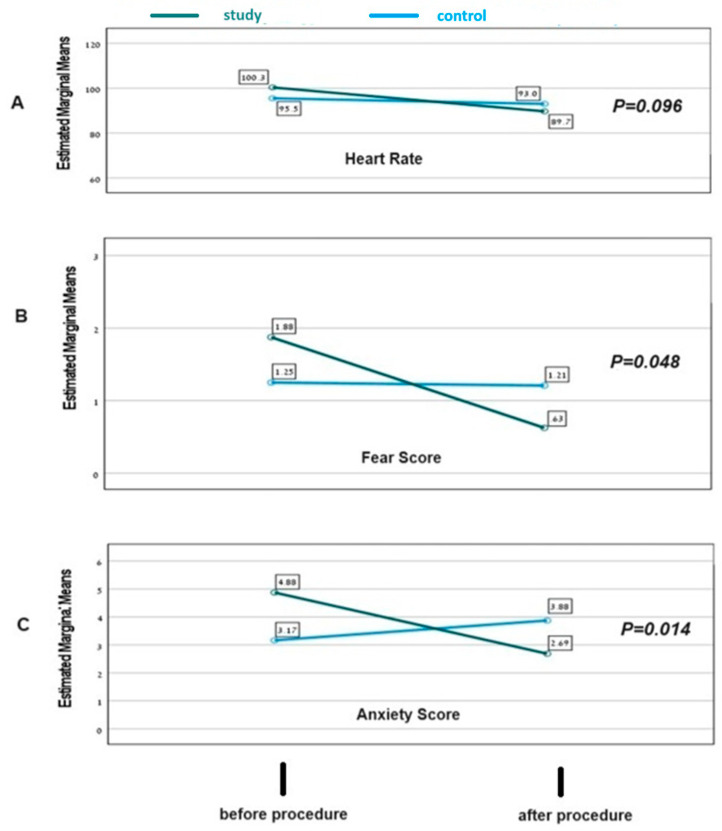
Repeated measure analysis for comparing changes in physiological and emotional parameters before and after the procedure in the control group and study group. (**A**) Comparison of changes in heart rate; (**B**) comparison of changes in fear scores; (**C**) comparison of changes in anxiety scores.

Repeated measure analysis was performed to compare changes in heart rate, and in fear and anxiety scores following procedures between the groups. This analysis demonstrated significant differences between participants who used the goggles and control. In participants who used goggles, there was a significant decrease in fear and anxiety scores after the procedure as compared to control (*p* = 0.048 and *p* = 0.014, respectively) (Figure 2).

### 3.4. Prediction of Physiological and Emotional Parameters According to Demographic/Clinical/Procedure-Related Characteristics

The general linear model revealed no associations of any of the demographic, clinical, or procedure-related characteristics with any of the physiological or emotional parameters either prior to or after the procedures were performed. Similarly, a multivariate test performed on repeated measure analysis to identify predictors of changes in physiological and emotional parameters following procedures did not find any predictors of changes in these parameters. The characteristics tested were age, sex, diagnosis, type of procedure, time from admission to procedure, and number of similar procedures performed prior to the study.

## 4. Discussion

In this study, we demonstrated the efficacy of ID-approved VR goggles for reduction of fear and anxiety associated with drain removal, wound treatment, blood draws, and peripheral vein-line insertion in pediatric surgical patients. On the other hand, we also found that pre-procedural anxiety was increased among children who used VR goggles. We found that post-procedural levels of fear, pain, and anxiety were lower in patients who used VR goggles as compared to control; however, the differences were not statistically significant.

The significant reduction in fear and anxiety scores among participants who used VR goggles suggests that the use of VR technology during common procedures performed in the pediatric surgical ward can be effective in mitigating negative emotional responses in these patients. Similarly, numerous previous studies reported the efficacy of VR technology in reduction of fear, anxiety, and pain in children undergoing various procedures [1,2,3,4,5,6,24]. Nevertheless, we did not find a significant advantage of VR technology when comparing post-procedural physiological and emotional parameters to control. In the literature, only a few studies report lack of benefit of VR technology or mixed and even worse outcomes when compared to standard care [25] or when compared to non-VR distraction methods [16,24,26,27,28,29]. When compared to nurse-provided standard care for adolescents undergoing burn wound care, different parameters other than interactive VR were found to influence pain intensity perception during treatment. These parameters included pre-procedure state and trait anxiety, pre-wound care pain score, time from original burn to clinic burn care treatment, and length of wound care treatment. The authors concluded that knowing patients’ needs, desires, and temperaments along with the specifics about the healthcare procedures were critical to formulating individualized care plans that may or may not include VR [25]. In another study, when compared to a similar tablet video, heart rate increase from pre-IV insertion to during IV insertion was significantly higher for those in the VR group. This result was observed despite a higher level of immersion in the distracting intervention in the VR group. In this study, there were no statistically significant differences between the VR and the tablet attention control groups. The authors did not provide an explanation for the increase in heart rate in the VR group, and noted that comparing VR to attention control likely yields lower effect sizes than comparing VR to no distraction [16]. Bagher did not find differences in heart rate and anxiety scores in children during dental treatment who were treated with VR movie versus children who watched a video cartoon over a regular screen. Nevertheless, salivary cortisol levels were signifantly lower in children in the VR group [29]. When 3D VR was compared to 2D distraction during the induction of anaesthesia, anxiety levels were similar, although children using 3D VR were less likely to have a perfect induction than those using 2D [24]. A large metanalysis on the use of VR technology for dental procedures in children, which included 11 studies, found significantly better outcomes in subjects who were exposed to VR during the dental procedure [30]. Another such metanalysis [31] found that nine studies reported reduced dental fear and anxiety, pain, and heart rate with the use of VR with only one study demonstrating exacerbation in children’s anxiety. The authors postulated that wearing a large VR headset over the face could lead to reduction in the visual field, causing loss of control and potentially exacerbating children’s anxiety.

Our data pointed to an increase in pre-procedural anxiety among children who used VR goggles. This can be attributed to the fact that pre-procedural physiological and emotional evaluation was performed after allocation of participants into the study versus control groups at the same time as preparation of the VR headset next to the children. It is possible that the recognition of being introduced to a new device, and even viewing the staff preparing it, added to the anxiety burden. It is possible that if the evaluation of pre-procedural parameters had been performed prior to group allocation, as in some other studies [16,24], we would not have observed pre-procedural differences between the groups. In addition, it is possible that if the participants had been given more time to become comfortable in the VR environment before the trial, this could have decreased anxiety associated with VR use. For example, in a study where the medical procedure (cast removal) was started only after the patients were given time to try the game and be familiar with it, post-procedural anxiety was significantly decreased in the VR group [1]. The few studies which indicated that application of VR goggles was associated with mixed and even negative effects on emotional status might in fact represent a pre-procedural added emotional burden which outweighs the benefits of the VR technology. This possbile emotional burden attributed to interaction with the VR gear was not measured in most studies [26,28,32]. Felemban, on the other hand, demonstrated that when VR goggles were applied in children undergoing injection of buccal local anaesthesia, heart rate checked at five time points was higher when compared to the control group. These time points included baseline and the time point when video was already turned on prior to injection [27].

In our intention to treat group, we encountered a group of participants who removed the goggles after initially receiving them. This happened despite assent given from children and consent given from parents for participation in the study. Possibly, patients’ clear wish to use the goggles upon offer rather than assent and parents’ consent may decrease this phenomenon and possibly mitigate the negative effect of the goggles on pre-procedural anxiety levels. It is also possible that some children with high trait anxiety are unable to completely immerse themselveves in a VR world [33]. We did not measure trait anxiety. We found only a few previous reports on children who were originally randomized to wear VR goggles after assent and consent were given, but then eventually refused to wear the VR equipment or removed them [3,6,18,30]. For example, Waltner-Larsen noted that 3 patients out of 32 who were allocated to receive VR googles disliked the VR game and the setup, leading to discontinuation [3]. Litwin noted that 1 out of 32 children who was allocated to receive VR goggles withdrew prior to the procedure, and that 2 patients declined to participate in the study prior to allocation because they were anxious about the procedure [16]. Chang noted 1 patient among 15 allocated to receive VR googles who refused to don the VR equipement [7]. It is possible that this phenomenon is under-reported as these children might have been excluded from analysis in other studies. It is also important to note a study which investigated the desire of participants who used VR to use them again. This study found that only 86.7% expressed such desire, while 2.2% did not want to use VR goggles in the future and 11.1% were not sure [1]. These findings might imply that some children do not enjoy using VR goggles despite keeping them on throughout the procedure.

We found that age, gender, diagnosis, length of stay prior to the experiment, type of procedure, and prior experience were not correlated with emotional status nor with emotional changes. Therefore, the higher proportion of females in the study group probably did not affect the results. The data in the literature are diverse. Felemban found that both female participants and younger children were more likely to report higher pain scores during local buccal anaesthesia administration regardless of the type of distraction used [32]. This is in contradistinction to a report by Attar, who found no differences in anxiety scores between males and females [28], and Chang, who found no differences in emotional scores associated with children’s age [7]. Jeffs found that pain following burn wound care treatment was associated with time from original burn to burn care treatment, as well as length of wound care treatment [25].

The type of goggles used in our study might have also had an effect on the results. There are various types of VR goggles and technologies that have been explored in research for medical and therapeutic purposes. The effectiveness of VR technology depends on factors such as design, comfort, and immersive experience. The VR goggles used in our study were mobile VR headsets, non-sponge-coated, to comply with infectious disease guidelines following the COVID-19 pandemic. These headsets use a smartphone inserted into a docking device as the screen and processor and are less comfortable and less powerful than sponge-coated systems. The use of the non-sponge-coated goggles was a necessary compromise to ensure ID safety. It is possible that the use of more comfortable goggles which offer better graphics would have led to better compliance and better results. In addition, other non-foam-coated systems that are not mobile mounted, that were not evaluated by us, could be superior to the system used in the study and still be compliant with ID requirements.

Our study has several limitations. First, our control group included standard care alone. Adding a second control group, as described by others [34] using other, non-immersive distractions, such as a telephone video, would have enabled a better evaluation of the possible unique contribution of immersive VR. Another comparison, to interactive VR gaming, could also add a better assessment of the best distraction method. Augmented reality, which is also less immersive than VR, and is believed by some authors to bridge some of the limitations of VR as a completely immersive experience, was also not tested. Secondly, baseline measurements were performed after allocation for study versus control groups. Additional pre-allocatioin measurements could have validated or contradicted the theoretical VR associated anxiety. In addition, we did not allow time for the participants to become confortable with the VR goggles prior to the pocedure, a step which might have decreased possible VR goggles-associated anxiety and even could have improved the effectiveness of the VR goggles. Better hardware and better software than those used in the study, a choice of different headset sizes, and non-cell-phone-mounted spongeless headsets could potentially improve the experience and the results. Adding evaluation of participants’ wish to use the goggles again could add a significant insight into the subjective experience. A larger sample size would have increased the statistical power of the results.

## 5. Conclusions

Using ID-approved VR goggles can significantly reduce fear and anxiety associated with procedures commonly performed in children treated in pediatric surgical wards, although post-procedural differences between VR and control were not found to be statistically significant. Baseline anxiety measured after allocation to use VR goggles was increased when compared to standard care. It is therefore possible that a higher benefit might be achieved if VR goggles were used only by children who express a clear wish to use them.

## Figures and Tables

**Table 1:** Clinical, demographic, and procedure-related characteristics of study and control groups.

	Control (N = 24)	Study (N = 16)	*p*-Value
**Age (years)**	11.6 ± 4.6	10.8 ± 4.2	0.56
**Sex**			
**Male**	17 (71%)	6 (37.5%)	**0.04**
**Female**	7 (29%)	10 (62.5%)	
**Diagnosis**			
**Appendicitis**	11 (46%)	6 (37.5%)	0.87
**Abdominal abscess**	5 (21%)	5 (31%)	
**Trauma**	4 (17%)	2 (12.5%)	
**Other**	4 (17%)	3 (19%)	
**Procedure**			
**D**	9 (38%)	7 (44%)	0.68
**IV**	14 (58%)	9 (56%)	
	1 (4%)	0	
**Time from admission to procedure (days)**	4.88 ± 3.9	5.5 ± 3.8	0.62
**Number of similar previous procedures**			
**0**	11 (46%)	9 (56%)	0.48
**1**	8 (33%)	5 (31%)	
**2**	1 (4%)	1 (6%)	
**3+**	4 (17%)	1 (6%)	

D—drain removal; IV—blood draw and/or insertion of peripheral IV cannula; W—wound care; in bold- *p* < 0.05

**Table 2:** Comparison of physiological and emotional parameters before and after procedures in the control and study groups.

Group	Parameter	Before Procedure	After Procedure	*p*
**Control**	Heart rate	95.48 ± 20.83	93 ± 23.29	0.33
	Fear	1.25 ± 1.03	1.21 ± 1.59	0.92
	Anxiety	3.17 ± 1.81	3.87 ± 3.21	0.55
**Study**	Heart rate	100.33 ± 17.98	89.67 ± 20.33	**0.036**
	Fear	1.87 ± 1.50	0.63 ± 1.09	**0.019**
	Anxiety	4.88 ± 3.12	2.69 ± 2.39	**0.024**

Bold- *p* < 0.05.

**Table 3:** Comparison of physiological and emotional parameters before and after procedure between the groups.

	Control (N = 24)	Study (N = 16)	*p*-Value
**Before procedure**			
Heart rate	95.5 ± 20.8	98.2 ± 19.8	0.69
Fear score	1 [0–2]	2 [0.25–3]	0.19
Anxiety score	3.17 ± 1.81	4.88 ± 3.11	**0.03**
**After procedure**			
Heart rate	93.0 ± 23.3	89.7 ± 20.3	0.68
Fear score	0 [0–2.75]	0 [0–1]	0.36
Anxiety score	3 [1–5.75]	2 [1–3.75]	0.29
Pain score	4.67 ± 3.48	4.31 ± 3.3	0.75

Bold *p* < 0.05.

## Data Availability

The data presented in this study are available on request from the corresponding author.
